# REV7 in Cancer Biology and Management

**DOI:** 10.3390/cancers15061721

**Published:** 2023-03-11

**Authors:** Yoshiki Murakumo, Yasutaka Sakurai, Takuya Kato, Hiroshi Hashimoto, Masaaki Ichinoe

**Affiliations:** 1 Department of Pathology, Kitasato University School of Medicine, 1-15-1 Kitasato, Minami-ku, Sagamihara 252-0374, Japan; 2 School of Pharmaceutical Sciences, University of Shizuoka, 52-1 Yada, Suruga-ku, Shizuoka 422-8002, Japan

**Keywords:** cancer management, cell cycle, DNA repair, inhibitor, protein–protein interactions, REV7

## Abstract

**Simple Summary:**

REV7 is a multifunctional protein involved in various biological processes including DNA damage response and mutagenesis, cell cycle regulation, primordial germ cell maintenance, and cancer cell biology. Although human REV7 was originally discovered as a homologous molecule to a mutagenic protein, Rev7, and a spindle assembly checkpoint protein, Mad2, in the yeast *Saccharomyces cerevisiae*, investigations of vertebrate REV7 identified several novel biological networks surrounding REV7. In addition, studies using human cancer tissues and cancer cell lines revealed the significance of REV7 in cancer biology, which makes REV7 an attractive target molecule in cancer management. This review focuses on the functions of REV7 in human cancers and discusses the utility of REV7 in cancer management.

**Abstract:**

DNA repair and cell cycle regulation are potential biological fields to develop molecular targeting therapies for cancer. Human REV7 was originally discovered as a homologous molecule to yeast Rev7, which is involved in DNA damage response and mutagenesis, and as the second homolog of yeast Mad2, involved in the spindle assembly checkpoint. Although REV7 principally functions in the fields of DNA repair and cell cycle regulation, many binding partners of REV7 have been identified using comprehensive analyses in the past decade, and the significance of REV7 is expanding in various other biological fields, such as gene transcription, epigenetics, primordial germ cell survival, neurogenesis, intracellular signaling, and microbial infection. In addition, the clinical significance of REV7 has been demonstrated in studies using human cancer tissues, and investigations in cancer cell lines and animal models have revealed the greater impacts of REV7 in cancer biology, which makes it an attractive target molecule for cancer management. This review focuses on the functions of REV7 in human cancer and discusses the utility of REV7 for cancer management with a summary of the recent development of inhibitors targeting REV7.

## 1. Introduction

*REV7* was originally identified as a critical gene of the yeast *Saccharomyces cerevisiae* (*S. cerevisiae*), *rev7*, a “reversionless” mutant that shows a less mutagenic phenotype after UV irradiation [1,2,3]. The *rev7* mutant shows a phenotype that is closely similar to another reversionless mutant, *rev3*, and both genes are required for misrepair mutagenesis [1,2]. It was soon revealed that *S. cerevisiae* Rev7 and Rev3 proteins form a complex, resulting in mutagenic DNA polymerase ζ (Polζ), in which Rev3 is the catalytic subunit and Rev7 is the accessory subunit [4]. Polζ is involved in translesion DNA synthesis (TLS), which is a DNA damage-tolerance mechanism that synthesizes DNAs through damaged sites of the template DNAs [5,6]. Human *REV7* (also known as *MAD2L2*, *MAD2B*, *MAD2β,* and *FANCV*) was identified as a homologous gene to spindle assembly checkpoint gene *MAD2*, as well as to yeast *REV7*; therefore, research into *REV7* was first progressed in the fields of TLS and cell cycle checkpoint [7,8,9]. Then, *REV7* was studied in mice, *Xenopus*, *Drosophila*, *Neurospora*, plants (*Arabidopsis*), and chickens, as well as in humans and *S. cerevisiae* [10,11,12,13,14,15,16]. Identification of new binding partners of REV7 with comprehensive analyses using the yeast two-hybrid assay and GST pull-down or co-immunoprecipitation assays, followed by mass spectrometry analyses, contributed to determining its significance in various biological processes other than TLS and cell cycle checkpoint. These include double-strand break (DSB) repair, homologous recombination (HR), telomere maintenance, the Fanconi anemia (FA) pathway, gene transcription, epigenetics, primordial germ cell (PGC) survival, neurogenesis, intracellular signaling, and microbial infection. In addition, investigations using human cancer tissues and cancer cell lines revealed the significance of REV7 in cancer development, proliferation, and progression, as well as its utility in cancer management. Recently, several excellent reviews describing the biological functions of REV7 have been published [17,18]; therefore, this review focuses on the significance of REV7 in cancer development, progression, and treatment, with an overview of REV7 functions associated with cancer biology.

## 2. Protein Structure of REV7

**Figure 1:**
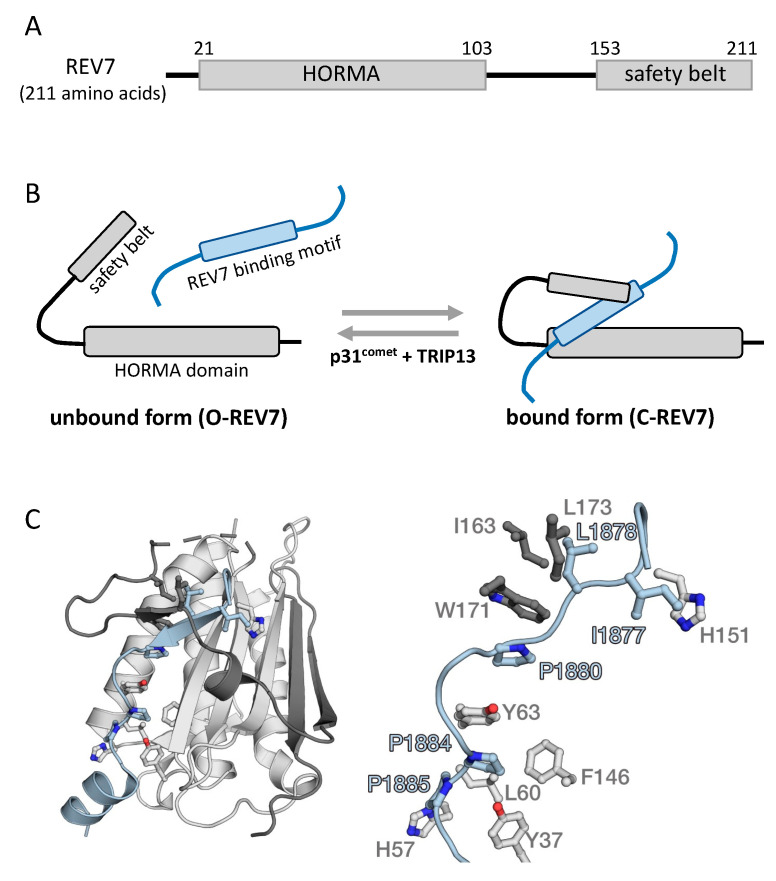
Structure of REV7 in complex with binding partners. (**A**) Domain structure of REV7. REV7 is composed of 211 amino acid residues, with a HORMA domain in its middle region and a “safety belt” region in its C-terminus [27]. (**B**) Bound and unbound forms of REV7. When REV7 unbinds its interaction partners, the safety belt structure is open (O-REV7), and when it binds its interaction partners, the safety belt structure is closed (C-REV7). The conformational change from C-REV7 to O-REV7 is catalyzed by p31^comet^ and TRIP13. TRIP13, thyroid hormone receptor-interacting protein 13. (**C**) Structure of human REV7(R124A) in complex with a human REV3 fragment including residues 1877–1885 (PDB entry 3ABD). REV7 and REV3 are shown in gray and light blue, respectively. The C-terminal region of REV7 is shown in a darker color. A detail of the interactions between REV7 and REV3 is shown in the right panel. I1877, L1878, P1880, P1884, and P1885 of REV3 in the consensus sequence for REV7 binding, ΦΦxPxxxpP, are shown as ball-and-stick models and labeled. Amino acid residues of REV7 involved in the interactions with the REV3 fragment are also shown as ball-and-stick models and labeled.

The human REV7 protein is composed of 211 amino acid residues, with a HORMA domain in its middle region but without any catalytically active domains (Figure 1A) [19]. The HORMA domain was originally proposed for its sequence similarity in yeast Hop1, Rev7, and Mad2 proteins [20]. The HORMA domain is supposed to be a domain for protein–protein interactions, and several HORMA family proteins have been identified in humans, including REV7 in DNA repair, MAD2 and p31^comet^ in spindle assembly checkpoint, HORMAD1 and HORMAD2 in meiosis, and ATG13 and ATG101 in autophagy [19,20,21,22,23,24,25,26]. The HORMA domain is important for the interactions of REV7 with many effector proteins. The three-dimensional structure of REV7 in complex with the effector proteins demonstrates that REV7 possesses a “safety belt” region in its C-terminus, which plays a critical role in catching the interacting peptides (Figure 1A) [25,27,28]. When REV7 binds to other proteins, the safety belt structure closes (C-REV7), and when it unbinds from the proteins, the safety belt structure opens (O-REV7) (Figure 1B). The crystal structure of REV7 interacting with a REV3 fragment demonstrates that the C-terminal region of REV7 wraps the REV3 fragment, thereby resulting in C-REV7 (Figure 1C). The interactions with binding partners are quite important for the function of REV7; however, what triggers the conformational change between C-REV7 and O-REV7 and controls its activity is still under investigation. Recently, it was revealed that p31^comet^ and thyroid hormone receptor-interacting protein 13 (TRIP13), an AAA+ ATPase, physically interact with REV7 and catalyze the conformational change from C-REV7 to O-REV7, controlling the functional activity of REV7 (Figure 1B) [29,30]. REV7 has numerous binding partners in various biological fields, and the consensus sequence for REV7 binding is proposed as ΦΦxPxxxpP, in which Φ is aliphatic, x is any residue, and proline p is less significant than proline P [31]. For example, REV3 has two REV7 binding sites: ILKPLMSPPS at amino acids 1877–1886 and VIMPCKCAPSR at amino acids 1993–2003, both of which are consistent with the REV7 consensus binding sequence [32].

## 3. Role of REV7 in Translesion DNA Synthesis and DNA Damage-Induced Mutagenesis

TLS is a DNA damage-tolerance mechanism that saves cells from the adverse consequences of replication arrest raised by DNA damage [5,33]. DNA damage is largely repaired by the DNA repair system before replication, but it sometimes escapes from the DNA repair network and remains unrepaired. When the replication fork encounters an unrepaired lesion on the template DNA, replicative DNA polymerase δ/ε (Polδ/ε) cannot replicate DNA through the lesion. In this situation, several specialized low-fidelity DNA polymerases proceed with the replication throughout the lesion on behalf of Polδ/ε, which is called translesion DNA synthesis (TLS). Finally, DNA replication completes through the lesion without removing the damage. However, in compensation for saving cells from replication fork collapse, error-prone TLS sometimes incorporates an incorrect nucleotide opposite the DNA lesion, resulting in the introduction of mutations.

**Figure 2:**
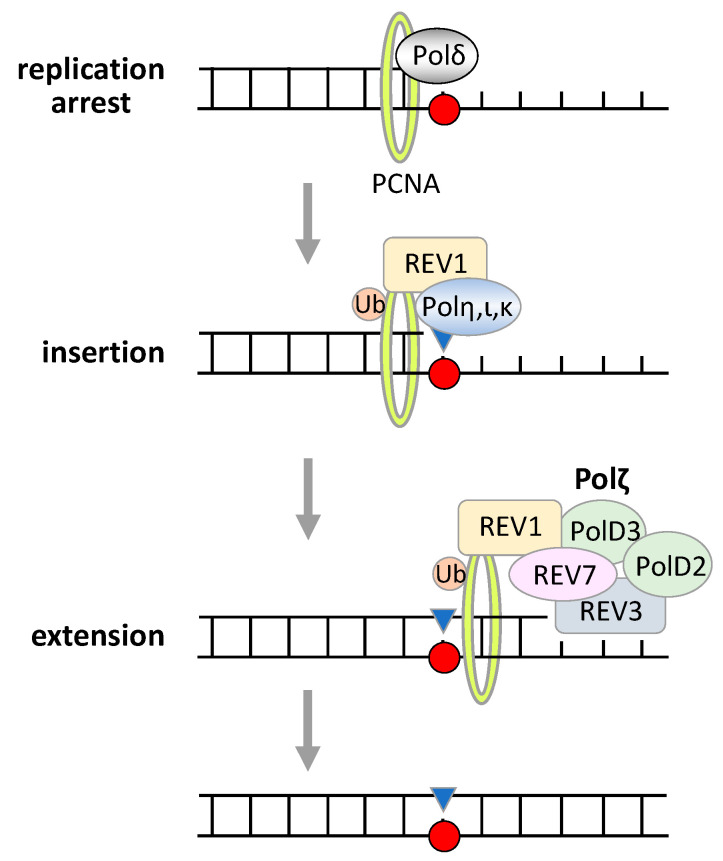
Mechanism of translesion DNA synthesis (TLS). When the replication fork stops at a DNA lesion (replication arrest), REV1 binds to monoubiquitinated proliferating cell nuclear antigen (PCNA) at the lesion and recruits inserter polymerases Polη, ι, and κ to the damaged site to incorporate a nucleotide opposite the damaged site (insertion). Then, REV1 recruits Polζ via interaction with REV7 or PolD3, and Polζ proceeds with the subsequent extension from the improperly paired DNA end (extention). Finally, TLS completes without removing the damage. Ub, ubiquitin.

REV7 is a subunit of Polζ, a low-fidelity TLS polymerase, which is composed of REV3, REV7, Pol31, and Pol32 in the yeast *S. cerevisiae*, and of REV3, REV7, PolD2, and PolD3 in humans (Figure 2) [4,34,35,36,37]. REV3 possesses polymerase activity, while the other three proteins are non-catalytic subunits required for the efficient TLS activity of Polζ [38]. The biological activity of Polζ in TLS has been mainly studied in the yeast *S. cerevisiae* because human REV3 is a huge protein of approximately 350 kDa and is difficult to be analyzed in vitro [9,39,40,41,42]. The TLS activity of Polζ in synthesizing DNA past a thymine–thymine *cis-syn* cyclobutane dimer was first demonstrated in the yeast *S. cerevisiae* [4]; it was revealed that the TLS activity of yeast Polζ is very limited and that another TLS polymerase is required to complete TLS in cooperation with Polζ [43,44,45,46]. Now, the consensus about the mechanism of TLS is that when the replication fork stops at a DNA lesion, an “inserter” TLS polymerase, such as Y family DNA polymerase Polη, ι, κ, or REV1, incorporates a nucleotide opposite the damaged site, and then Polζ proceeds with the subsequent extension from the improperly paired DNA end as a “mispair extender” (Figure 2) [43,44,45,46]. This mechanism of TLS has been also achieved using human Polζ in cisplatin bypass [37].

The REV7 subunit of Polζ also interacts with REV1, which is required for the efficient TLS activity of Polζ (Figure 2) [47,48,49]. REV1 also binds to other inserter polymerases Polη, ι, κ, and the PolD3 subunit of Polζ, and REV1 itself possesses deoxycytidyl transferase activity; thus, REV1 is thought to be a hub protein of the TLS machinery [50,51,52]. When the replication fork encounters damage on template DNA, REV1 binds to monoubiquitinated proliferating cell nuclear antigen (PCNA) at the lesion and recruits inserter polymerases Polη, ι, and κ to the damaged site, as well as Polζ via interaction with REV7 or PolD3, although the mechanistic details remain controversial (Figure 2) [6,53,54,55,56]. The transcription factor TFII-I also binds to PCNA and REV7 and promotes TLS by connecting Polζ with PCNA [57]. Conversely, the physical interaction of p31^comet^ and TRIP13 with REV7 facilitates the dissociation of REV7 from REV3 by promoting a conformational change from C-REV7 to O-REV7, resulting in inhibition of TLS activity of Polζ [29,30].

Polζ, together with REV1, is responsible for spontaneous and DNA damage-induced mutagenesis events via error-prone TLS [58,59]. The involvement of REV7 in damage-induced mutagenesis has been demonstrated using yeast and mammalian cells. *S. cerevisiae rev7* mutants showed a deficiency in UV-induced reversion mutations [1,2], while depletion in *REV7* by RNA interference in human fibroblasts or nasopharyngeal carcinoma cells decreases UV-induced or chemotherapy-induced mutation frequencies of *HPRT*, respectively [60,61], indicating that REV7 is involved in DNA damage-induced mutagenesis.

## 4. REV7 in Other DNA Repair Pathways

**Figure 3:**
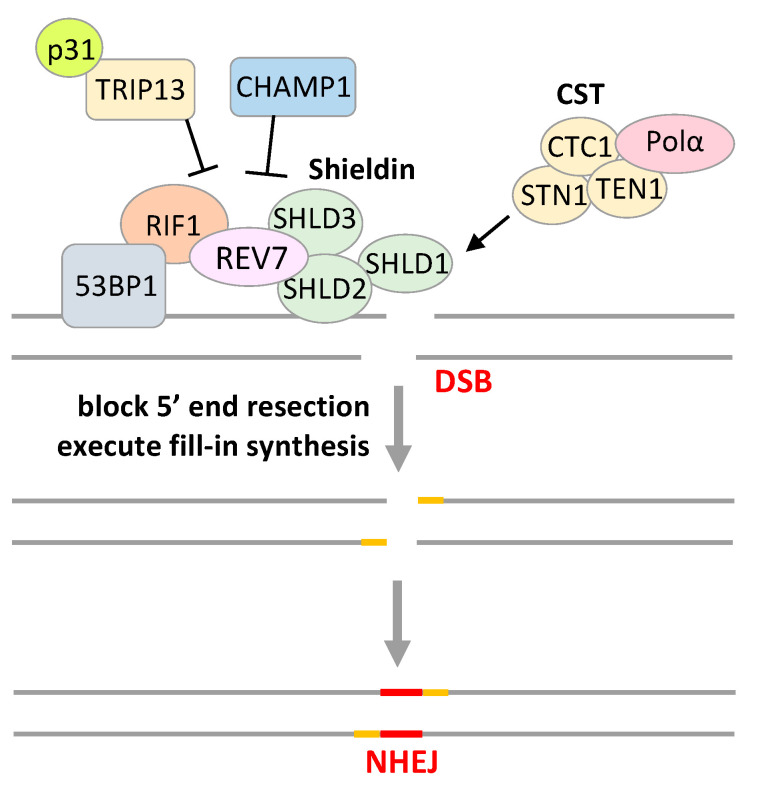
Involvement of REV7 in double-strand break (DSB) repair. At a DSB lesion, 53BP1–RIF1 recruits the Shieldin complex (SHLD1, SHLD2, SHLD3, and REV7) to block 5’ end resection, and Shieldin also recruits the CST complex (CTC1, STN1, and TEN1) together with DNA polymerase α (Polα) to execute fill-in synthesis, resulting in the inhibition of HR and the facilitation of non-homologous end joining (NHEJ). CHAMP1 binds to REV7 promoting the decreased level of the Shieldin complex, and p31^comet^–TRIP13 binds to REV7 in the Shieldin complex and promotes its dissociation from SHLD3 by inducing O-REV7 from C-REV7, resulting in a reduction in the Shieldin complex activity.

Recent progress in REV7 research revealed the importance of REV7 function in the DNA damage response other than TLS. *BRCA1*-mutated, HR-deficient breast or ovarian cancer cells are sensitive to poly(ADP-ribose) polymerase (PARP) inhibitors, and resistance to PARP inhibitors in *BRCA1*-mutated cells is induced by restoration of HR, which is triggered by REV7 depletion [62]. REV7 is recruited to DSBs under the control of TP53-binding protein 1 (53BP1) and RAP1 interacting factor 1 (RIF1), and inhibits 5’ end resection at DSB sites, resulting in the inhibition of HR and the facilitation of non-homologous end joining (NHEJ) (Figure 3). REV7 loss restores the 5’ end resection by CTBP-interacting protein (CtIP) at DSBs, which causes NHEJ inhibition and HR restoration, resulting in PARP inhibitor resistance [62]. In addition, REV7 is also recruited to uncapped telomere ends and inhibits 5’ end resection at telomere ends, facilitating NHEJ-mediated telomere fusion [63]. REV7 depletion promotes 5’ end resection and 3’ overhang at uncapped telomere ends, preventing telomere fusion [63]. Both studies discovered an important biological function of REV7 in DSB repair choice. At DSB lesions or uncapped telomere ends, 53BP1 is recruited with RIF1 under ataxia telangiectasia mutated (ATM)-or RAD3-related (ATR) signaling, and then 53BP1–RIF1 recruits the Shieldin complex, composed of C20orf196 (SHLD1), FAM35A (SHLD2), CTC-534A2.2 (SHLD3), and REV7, which acts at DSB lesions to block 5’ end resection and 3’ single-stranded overhang generation [64,65,66,67,68]. Shieldin also recruits CST complex, composed of CTC1, STN1, and TEN1, together with DNA polymerase α (Polα) to execute fill-in synthesis, resulting in the inhibition of HR and facilitation of NHEJ (Figure 3) [69,70]. The 53BP1-recruited Shieldin complex is also required for immunoglobulin class-switch recombination by NHEJ [64,65,66,67,68,69,70]. Inhibitory effects on REV7 are provoked by chromosome alignment-maintaining phosphoprotein 1 (CHAMP1) and p31^comet^–TRIP13. CHAMP1, which is involved in microtubule organization, binds to REV7 and reduces the Shieldin complex activity to promote 5’ end resection and HR repair at DSBs [71,72]. p31^comet^-TRIP13 binds to REV7 in the Shieldin complex and promotes its dissociation from SHLD3 by inducing O-REV7 from C-REV7, resulting in a reduction in the Shieldin complex activity (Figure 3) [29,30].

Inconsistently, the human Polζ/REV1 complex is involved in HR repair. REV1-, REV3-, or REV7-depleted HeLa cells display enhanced chromosomal instability induced by ionizing radiation (IR) due to a deficiency in HR repair at IR-induced DSBs [73]. Similarly, chicken DT40 cells lacking *REV1*, *REV3*, and *REV7* show suppressed levels of sister chromatid exchange (SCE), suggesting HR insufficiency, although DT40 cells with every single deletion show elevated levels of SCE [16]. These findings seem incompatible with the role of REV7 in the Shieldin complex, indicating that complicated roles of REV7 are involved in the DNA damage response via various binding partners.

*REV7* also belongs to the FA genes, whose germline mutations cause an autosomal recessive disorder characterized by congenital abnormality, chromosomal instability, bone marrow failure, and cancer predisposition [74]. *REV7* with a biallelic inactivating mutation encoding REV7-V85E mutant protein was identified in an FA patient. Patient-derived cells showed undetectable levels of REV7 with normal transcript levels, suggesting destabilization of the mutant protein, and displayed phenotypes of interstrand crosslink repair failure, which was restored by the expression of wild-type REV7, indicating that *REV7* is the 21st FA gene, termed FANCV [74].

Accordingly, REV7 plays important roles in the replication stress response, which is associated with cancer biology [75]. REV7 is involved in both TLS and DSB repair, the former solves replication stress raised by various types of DNA damage by bypassing the lesion, and the latter solves replication stress raised by DSBs by facilitating NHEJ or HR. REV7 is also involved in the FA DNA repair pathway, which solves the replication stress of DNA interstrand crosslink. Thus, REV7 dysfunction should be associated with cancer biology, which will be discussed later.

## 5. REV7 in Cell Cycle Regulation and Gene Transcription

**Figure 4:**
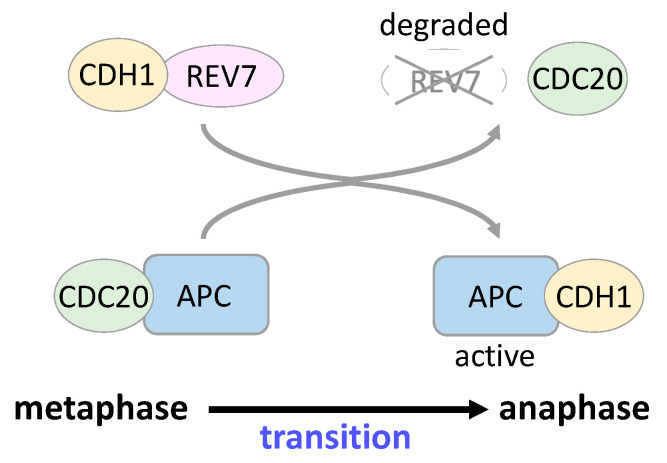
REV7 involvement in the mitotic checkpoint. REV7 binds to CDH1 to sequester it from APC in prometaphase, and APC–CDC20 degrades REV7 in early anaphase; released CDH1 activates APC, leading to a transition from metaphase to anaphase.

Human REV7 is a homologous molecule of *S. cerevisiae* Mad2, a spindle assembly checkpoint protein, and interacts with human MAD2 [9]. *Xenopus* Rev7 directly binds to anaphase-promoting complex (Apc) activator Cdc20 homolog 1 (Cdh1) and inhibits the E3 ubiquitin ligase activity of Cdh1–Apc [10,11]. *Xenopus* Rev7 also binds weakly to another Apc activator, Cdc20, and inhibits Cdc20–Apc activity [11]. Injection of *Xenopus* Rev7 into one cell of a two-cell *Xenopus* embryo causes cell cycle arrest in the injected cell [10]. Human REV7 binds to CDH1 to sequester it from APC in prometaphase, and REV7 is degraded by APC–CDC20 in early anaphase; released CDH1 activates APC, leading to a transition from metaphase to anaphase (Figure 4) [76]. REV7 is localized around the metaphase plates and mitotic spindles, and its depletion causes cell cycle arrest in the G2/M phase, exhibiting abnormal spindle formation and chromosomal misalignment [77]. In addition, REV7 interacts with the Ras-related nuclear protein (RAN) small GTPase, Clathrin light chain A, and CHAMP1, all of which play important roles in microtubule organization and proper progression of the G2/M phase [28,76,78,79]. These findings indicate the involvement of REV7 in cell cycle regulation.

REV7 interacts with the ELK-1 transcription factor, promoting its phosphorylation by c-Jun N-terminal protein kinase (JNK) mitogen-activated protein (MAP) kinase and facilitating its transcription activity following DNA damage [80]. REV7 binds to WNT signal transducer T cell factor 4 (TCF4) and blocks TCF4-mediated transactivation of downstream target genes such as *SLUG* in colon cancer cells. REV7 depletion suppresses E-cadherin expression via upregulation of SLUG and promotes TCF4-mediated epithelial–mesenchymal transition (EMT) in colon cancer cells [81].

## 6. REV7 in Cancer

### 6.1. Possible Role of REV7 in Cancer Development

Cancer development is mainly caused by mutations in cancer-related genes or by epigenetic regulation; thus, molecules involved in mutagenesis are thought to be associated with cancer development. TLS is a DNA damage-tolerance mechanism and is a cause of DNA damage-induced and spontaneous mutations. Polζ is involved in most of the TLSs, including error-free and error-prone TLSs, playing an essential role in TLS [19,33]. The frequency of UV-induced mutations in human fibroblast cells with siRNA-mediated depletion in *REV7*, as well as *REV3* and *REV1*, is significantly decreased compared with control cells, which may be caused by abrogation of Polζ activity [39,60,82]. Suppressed spontaneous and damage-induced mutagenesis, increased chromosomal aberrations, and H2AX phosphorylation also occur in cancer cells with *REV7* depletion after treatment with various DNA-damaging reagents, indicating the involvement of REV7 in damage-induced mutagenesis in cancer cells [61]. Considering the biological role of REV7 in DNA damage-induced mutagenesis, dysregulated REV7 expression is supposed to promote cancer development via the introduction of mutations in cancer-related genes or in regions of epigenetic regulation, although there is no direct evidence showing the relationship between REV7 expression and cancer development thus far. Conversely, *Rev7^C70R/C70R^* mutant mice, in which mutant REV7 is unable to interact with REV3, causing germ cell aplasia after birth in both males and females, develop tubulostromal adenomas in the ovary, in which an increase in gonadotropin levels and accumulation of DNA damage may contribute to tumor development [83,84]. Notably, *REV7* is a causative gene of FA, which shows a predisposition for the development of hematological malignancy [74].

### 6.2. Clinical Significance of REV7 in Cancer

The significance of REV7 expression has been studied using clinical materials from various cancer tissues. Although REV7 expression is low in most normal human tissues except for the testis, its expression is relatively high in various human tumor tissues, including colon, ovarian, breast, esophageal, lung, and skin cancers, gliomas, diffuse large B cell lymphomas, and testicular germ cell tumors (TGCTs) [85,86,87,88,89,90,91,92,93]. REV7 expression levels are correlated with cell proliferation ability represented by Ki-67 labeling indexes in small cell lung carcinomas and malignant melanomas, with metastases in breast and lung cancers, with tumor thickness in malignant melanomas, and with tumor sizes of gliomas [89,92,93,94,95]. There is a significant association between high levels of REV7 expression and poor prognosis in several tumors, including colon, breast, lung, gastric, and advanced ovarian cancers, diffuse large B cell lymphomas, and advanced bone and soft tissue sarcomas, suggesting the utility of REV7 as a biomarker for cancer prognosis [85,87,88,89,94,96,97]. The mechanisms for the upregulation of REV7 in tumor tissues have not been fully elucidated, although zinc finger protein with KRAB and SCAN domains 3 (ZKSCAN3), an oncogenic transcription factor, acts as a transcriptional repressor of *REV7* in cultured tumor cells [98]. The contribution of REV7 to poor prognosis in various malignancies may be explained by the following: (1) REV7-high tumors exhibit the potential for high proliferation and tend to be more advanced [89,92,93,94,95]; and (2) REV7 expression affects cell mobility, intracellular signaling, EMT, and sensitivity to chemoradiotherapy, which will be discussed later. In support of this, a clinical study on advanced bone and soft tissue sarcomas demonstrated that REV7 expression is significantly high in non-responders to trabectedin and olaparib combination therapy [97]. A genetic polymorphism of *REV7* rs746218GG is also associated with shorter progression-free survival in patients with lung cancer who have received platinum-based chemotherapy, probably due to affecting the expression of REV7 [99]. In contrast, high expression of REV7 is associated with favorable prognosis in colon cancer patients, and REV7 overexpression suppresses cell proliferation, migration, and clonogenicity in colorectal cancer cells via the degradation of nuclear receptor coactivator 3 (NCOA3), a transcriptional coactivator that interacts with REV7 [100].

### 6.3. REV7 in Cancer Cell Biology

The significance of REV7 in cancer cell biology has been studied using cultured cancer cells, most of which demonstrate the cancer-promoting effects of REV7, and a possible application of REV7 to cancer management has been proposed. Inactivation of *REV7* with siRNA-mediated knockdown or CRISPR/Cas9 system-mediated gene silencing in a variety of cancer cells promotes the inhibition of cell growth, mobility, invasion, clonogenicity, and EMT in vitro, indicating that REV7 expression affects the nature of cancer cells [87,89,90,91,92,93,94,95,96,99]. Hypersensitivity to DNA-damaging agents such as cisplatin, carboplatin, melphalan, chlorethamine, doxorubicin, mitomycin c, or γ-irradiation, but not to H_2_O_2_, 5FU, taxol, or vincristine, decreased spontaneous and damage-induced mutation frequency, accumulation of DSBs and increased chromosomal aberrations after DNA damage, and suppressed cisplatin-induced SCE are promoted by *REV7* depletion in nasopharyngeal carcinoma cells [61]. REV7 suppression also sensitizes malignant melanoma, TGCT, and colon cancer cells to cisplatin, doxorubicin, or oxaliplatin, and sensitizes gliomas and esophageal squamous cell carcinoma (SCC) cells to IR [86,90,91,101]. In addition, tumor-bearing mouse experiments with *REV7*-depleted cancer cells also demonstrated suppressed cell proliferation, increased apoptotic cells, and enhanced sensitivity to cisplatin in vivo in ovarian cancer and TGCT cells [87,91]. Resistance to 5FU or oxaliplatin correlates with the upregulation of REV7 and high TLS efficiency in colon cancer cells [102], and REV7 inactivation overcomes acquired resistance to cisplatin in TGCT cells and to 5FU and oxaliplatin in colon cancer cells in vitro and in vivo [91,102]. Consistently, the overexpression of REV7 promotes chemoresistance in lung cancer cells and radioresistance in esophageal SCC cells [90,94]. Mechanistically, REV7 activates intracellular signaling of the PI3K–AKT and RAS–MAPK pathways and promotes TGF-β1 expression, resulting in the acceleration of cell proliferation, migration, invasion, and EMT [89,93,95]. These results indicated that REV7 is involved in cell activity via modulating intracellular signaling and that its depletion promotes increased sensitivity to chemoradiotherapy, conceivably by abrogation of TLS-mediated DNA damage tolerance.

Several molecules mediating the tumor-promoting and chemoradioresistant effects of REV7 have been identified. REV7 interacts with peroxiredoxin 2 (PRDX2), an antioxidant protein, and disruption of REV7–PRDX2 interactions increases oxidative stress and DSB post-irradiation [90]. In lung cancer cells, REV7 promotes the expression of SLUG, which might be an effector molecule for the tumor-promoting effects of REV7 [94]. REV7 exhibits opposing effects on SLUG expression in colon cancer cells and lung cancer cells, the reason for which is not known [81,94]. Papillary renal cell carcinoma-associated protein (PRCC) interacts with REV7, promoting its translocation from the cytoplasm to the nucleus. The translocation is impaired by PRCCTFE3, which is a fusion protein of PRCC and the transcription factor enhancer 3 (TFE3), and is frequently observed in papillary renal cell carcinoma, resulting in mitotic checkpoint defects in tumor cells [103]. Another mechanism for the chemosensitizing effect of REV7 inactivation is cellular senescence. Tumor cells with REV7 deficiency exhibited enhanced sensitivity to cisplatin in a mouse model of non-small cell lung cancer (NSCLC), in which cellular senescence, but not apoptotic cell death, was induced by DNA damage, showing a flattened, vacuolized cell morphology and induction of senescence-associated β-galactosidase [104]. REV7 inactivation facilitates cisplatin sensitivity more than REV3 inactivation, suggesting that the chemosensitizing effect of REV7 inactivation is mediated not only by TLS defects but also by insufficiency of the other biological roles of REV7 [104].

### 6.4. Development of Inhibitors Targeting TLS Associated with REV7

Identification of small molecules to inhibit the TLS activity associated with REV7 has recently been attempted (Table 1). A small compound, JH-RE-06, has been identified using ELISA in the screening of small-molecule inhibitors targeting REV1–REV7 interactions [105]. REV7 interacts with the REV1 C-terminal domain (CTD) to form Polζ–REV1 complexes, and JH-RE-06 binds to the REV1 CTD to promote dimerization of the REV1 molecules, resulting in the disruption of REV1–REV7 interactions. The addition of JH-RE-06 to cell culture medium significantly sensitizes various human and mouse cancer cells to cisplatin or other DNA-damaging agents. The addition of JH-RE-06 also suppresses TLS over a cisplatin adduct and decreases the frequency of both spontaneous and cisplatin-induced mutations in HT1080 cells. Moreover, JH-RE-06 is also effective in vivo. The injection of cisplatin plus JH-RE-06 into subcutaneous xenografted tumors in tumor-bearing mouse models significantly suppressed tumor growth and improved their prognosis compared with the injection of saline, cisplatin, or JH-RE-06 alone, indicating that targeting REV1–REV7 interactions together with DNA-damaging treatment is an effective strategy for cancer therapy [105]. Unexpectedly, cancer cells treated with cisplatin plus JH-RE-06 do not have increased apoptotic cell death but exhibit an enhancement in senescence markers such as senescence-associated β-galactosidase and p21 expression, suggesting that the addition of TLS inhibitors to DNA-damaging chemotherapy alters the apoptotic cell death effects of chemotherapy to senescence-based anti-tumor effects [106]. It is very interesting that these senescence-based anti-tumor effects were also demonstrated following REV7 loss in an NSCLC mouse model [104].

Small-molecule compounds identified with the screening of molecules that inhibit the interaction of REV7(R124A) and REV3(1875–1895) exhibit inhibition of interstrand crosslink repair and have a chemosensitization effect [107]. The small molecules ZINC97017995 and ZINC25496030, identified in a screen for molecules interfering with the interaction between REV1 and the REV7 dimer, directly bind to the REV7 dimer and disrupt REV7–REV1 interactions in vitro, enhancing the cell death effect of cisplatin in lung cancer cell lines [108].

These findings, together with the results from other similar attempts targeting the REV1-CTD, indicate that targeting the REV1/Polζ-associated TLS with small-molecule inhibitors is a promising strategy for cancer therapy [109,110,111,112,113].

## 7. Conclusions

In the past decade, many REV7 functions have been discovered, and we now understand REV7 to be a multifunctional protein involved in not only TLS and cell cycle regulation but also in many biological fields. However, many questions remain to be answered about REV7. Although REV7 has numerous binding partners and controls their activities, it is not clear what factors trigger the transition from the open to closed forms of REV7, and what happens to the binding partners through interactions with REV7. Regarding cancer biology and management, it is not clear whether mutagenic TLS involving REV7 really contributes to de novo tumor development via the introduction of mutations. High levels of REV7 expression are detected in some malignant tumor cells, but we do not understand the mechanisms that control REV7 expression in vivo, which may serve as important information to block REV7 expression for molecular targeting therapy. Although small molecules targeting REV1–REV7 interactions are effective in vitro and in vivo, an inhibitor targeting the global activity of REV7 may become another attractive tool for cancer therapy, because REV7 functions not only in TLS but also in other tumor-promoting conditions. In addition, it is important to elucidate the roles of REV7 in developmental biology, especially in PGC survival and differentiation, which is not discussed in this review [83,114,115].

Although more than 20 years have passed since human REV7 was identified, the number of identified REV7 interaction partners is still growing, and the significance of mammalian REV7 has been expanding in various biological fields. As the multifunctionality of REV7 becomes evident, the biological studies of REV7 become more complicated. However, recent progress in basic and clinical cancer research reveals REV7 to be a possible predictive biomarker for the effectiveness of DNA-damaging chemotherapy or PARP inhibitors and for the prognosis of cancer patients; it is also an attractive molecular target for the enhancement of DNA-damaging chemotherapy. Future research on REV7 may provide a new tool to treat cancer.

## Figures and Tables

**Table 1:** List of inhibitors targeting REV1/Polζ-associated TLS.

Inhibitor	Screening Target	Mechanism	Effect	References
JH-RE-06	REV1 CTD-REV7 PPI	Promote REV1 dimerization Disrupt REV1-REV7 PPI	Inhibition of cisplatin-induced mutagenic TLS Enhancement of cisplatin, BPDE, 4-NQO, and MMS cytotoxicity in vitro Enhancement of cisplatin chemosensitivity in vivo	[105,106]
Compound 7	REV3-REV7 PPI	Disrupt REV3-REV7 PPI	Inhibition of interstrand cross-link repair Enhancement of cisplatin cytotoxicity in vitro	[107]
ZINC97017995, ZINC25496030	REV7 homodimer	Disrupt REV1-REV7 PPI	Enhancement of cisplatin and doxorubicin cytotoxicity in vitro	[108]
Thiophene, piperazine, piperidine, and aryl piperazine compounds	REV1 CTD-Polκ RIR PPI	Disrupt REV1 CTD-RIR PPI	Enhancement of cisplatin and UV cytotoxicity in vitro Inhibition of cisplatin-induced mutagenesis	[109,110]
Phenazopyridine compounds	REV1 CTD	Disrupt REV1 CTD-RIR PPI	Enhancement of cisplatin cytotoxicity in vitro	[111,112,113]

CTD: C terminal domain, PPI: protein–protein interaction, RIR: REV1-interacting region, TLS: translesion DNA synthesis, BPDE: benzo[a]pyrene diol epoxide, 4-NQO: 4-nitroquinolone 1-oxide, MMS: methyl methanesulfonate, UV: ultraviolet.
